# Unlocking the brain: Biodistribution insights into tween 80 nanoliposomes in healthy brains

**DOI:** 10.1016/j.ijpx.2026.100636

**Published:** 2026-08-13

**Authors:** Masoud Khatibi, Seyedeh Maryam Hosseinikhah, Seyedeh Hoda Alavizdaeh, Fatemeh Gheybi, Mahmoud Reza Jaafari, Zohreh Efati, Farzaneh Afshari, Samaneh Yaghoubi, Parviz Norouzi

**Affiliations:** a Nanotechnology Research Center, Pharmaceutical Technology Institute, Mashhad University of Medical Sciences, Mashhad, Iran; b Center of Excellence in Electrochemistry, Collage of Science, University of Tehran, Tehran, Iran; c Department of Pathology, Erasmus, MC, the Netherlands; d Pharmaceutical Nanotechnology Department, School of Pharmacy, Mashhad University of Medical Sciences, Mashhad, Iran; e Department of Medical Biotechnology and Nanotechnology, School of Medicine, Mashhad University of Medical Sciences, Mashhad, Iran; f Biotechnology Research Center, Pharmaceutical Technology Institute, Mashhad University of Medical Sciences, Mashhad, Iran; g Student Research Committee, Mashhad University of Medical Sciences, Mashhad, Iran; h Department of Pathology, Mashhad University of Medical Sciences, Mashhad, Iran

**Keywords:** Doxorubicin, Nanoliposome, Tween 80, Blood brain barrier

## Abstract

Limited drug penetration across the blood–brain barrier hinders brain tumor therapy. This study evaluated doxorubicin-loaded nanoliposomes with varying Tween 80 contents, investigating formulation characteristics, release profiles, cytotoxicity on cancer and healthy cells, and tissue distribution in healthy models. Nanoliposomes were prepared *via* thin-film hydration. Physicochemical properties, *in vitro* drug release at physiological and acidic pH, and cytotoxicity (MTT assay and IC_50_) on C6 cells and normal NIH 3 T3 cells (including empty-carrier biosafety) were assessed. *In vivo* tissue distribution and histopathological safety were also examined in mice. Formulations showed composition-dependent size and 3-month stability. Formulation F2 emerged as the lead candidate, exhibiting optimal size (142.4 nm), PDI (0.139), encapsulation efficiency (82.4%), and pH-responsive release (80–90% at pH 5.5 *vs.* <10% at pH 7.4). F2 maintained potent cytotoxicity on C6 cells (IC_50_: 0.44 μg/mL) while demonstrating high biosafety on normal NIH 3 T3 cells, showing >90% viability with empty blanks and a markedly higher IC_50_ (20.67 μg/mL) than free DOX. *In vivo*, F2 achieved superior brain accumulation at 24 and 48 h (2.61 and 2.78 μg/g; *p* < 0.05 *vs.* DOX) while reducing cardiac uptake (2.29 *vs.* 14.49 μg/g at 24 h). Formulation F2 balances colloidal stability, reduced cardiotoxicity, and superior brain accumulation, supporting its potential for neuro-oncology applications.

## Highlights

• Tween 80-modified liposomes exhibited distinct cytotoxicity in C6 cells.
• Doxorubicin release was accelerated under acidic tumor-like conditions.
• The liposomes enhanced brain accumulation of doxorubicin.
• Formulation with 0.25% Tween 80 showed a considerable brain accumulation.
• Nanoliposomes remained physically stable for at least 3 months.

## Graphical abstract

**Figure:**
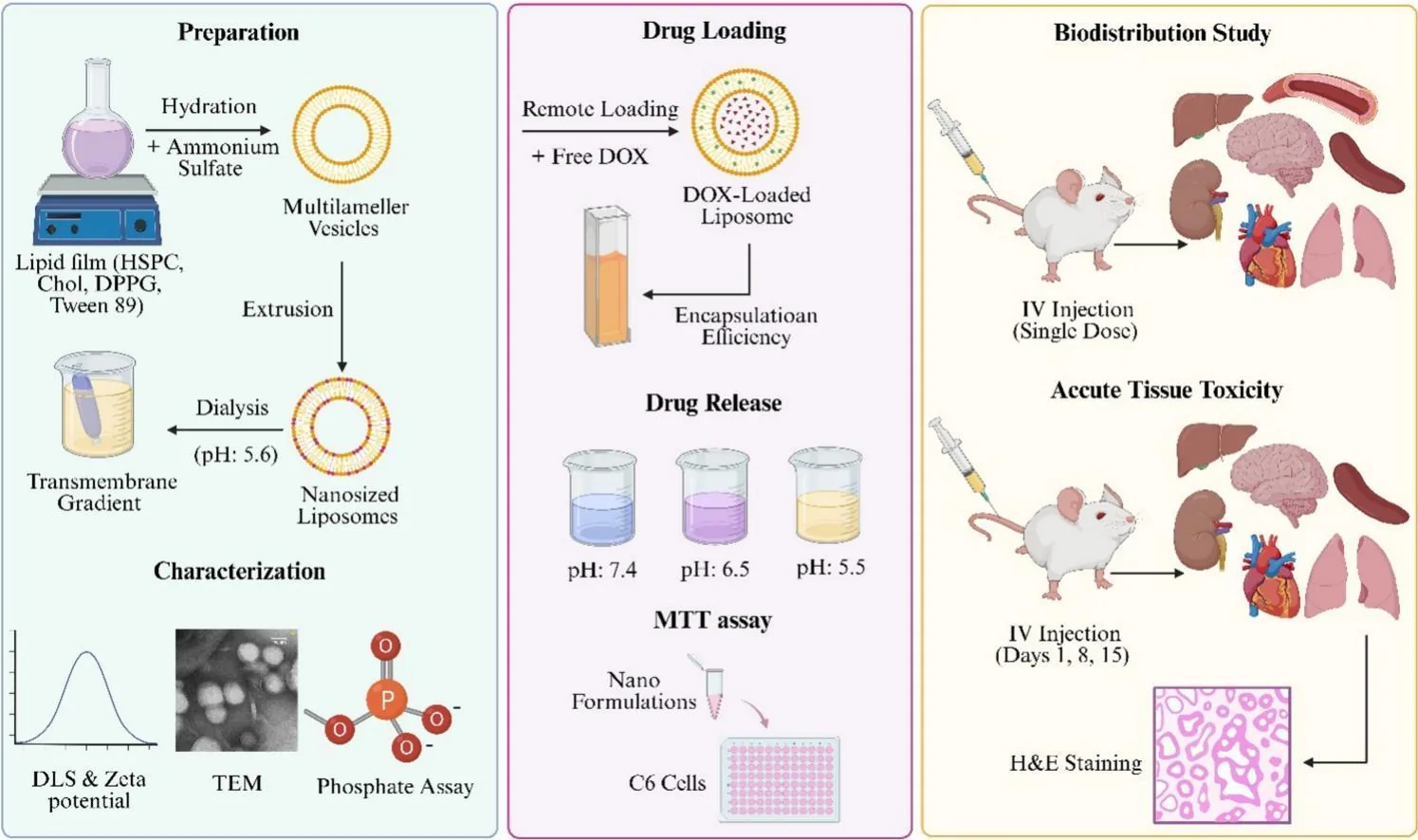

## 1 Introduction

Brain and central nervous system (CNS) tumors represent a diverse group of neoplasms that account for ≈1.35% of all cancers yet contribute disproportionately to mortality, representing 29.5% of cancer-related deaths worldwide (Pichaivel et al., 2022). Glial tumors form the major subset of malignant primary brain tumors, comprising around 81% of cases (Ardizzone et al., 2020). Among them, glioblastoma (GBM) is consistently identified as the most common malignant primary brain tumor**,** responsible for 14.5% of all CNS tumors and nearly half of all malignant CNS tumors (48.6%) (Grochans et al., 2022). Its incidence is estimated at 2–5 cases per 100,000 individuals per year in Europe and the United States, with higher prevalence in older adults and males (Bikfalvi et al., 2023). GBM is classified as a World Health Organization (WHO) grade IV diffuse astrocytic tumor and is biologically defined by aggressive proliferation, necrosis, and extensive microvascular remodeling (Ardizzone et al., 2020; Bikfalvi et al., 2023).

GBM therapy remains profoundly limited due to several biological and anatomical barriers (Archie et al., 2021; Proulx and Engelhardt, 2022). First, extensive inter- and intra-tumoral heterogeneity makes it difficult for any single treatment to eliminate all malignant subpopulations, enabling resistant clones to survive and drive recurrence (Rao et al., 2023). Drug efflux pumps, particularly ABCB1, ABCC1, and ABCG2, also expel therapeutic compounds from tumor cells, limiting intracellular drug accumulation and contributing to treatment failure (Robey et al., 2018; Sajid et al., 2023). Effective treatment of malignant brain tumors critically depends on a therapy's ability to cross the BBB, as the barrier's low permeability severely limits the application of many promising therapeutic agents and directly contributes to poor clinical outcomes(Mitusova et al., 2022).

Many clinically used anticancer agents show poor penetration across the BBB. For instance, doxorubicin (DOX) is approved for GBM treatment but exhibits low BBB permeability, and vincristine, although a potent microtubule inhibitor, “cannot penetrate the BBB” and therefore requires specific delivery strategies to reach brain tumors (Mitusova et al., 2022; Niazi, 2023). DOX is an anthracycline chemotherapeutic that exerts its anticancer effect (Kciuk et al., 2023). This multimodal mechanism underpins its broad clinical use as a first-line agent against a wide spectrum of solid tumors and hematologic malignancies, including breast cancer, lymphomas and sarcomas (Bhutani et al., 2025; Liao et al., 2021). Its clinical utility is further constrained by dose-dependent cardiotoxicity which limit the cumulative dose that can be safely administered (Rawat et al., 2021).

Conventional DOX formulations also have relatively short circulation times and unfavorable pharmacokinetics, whereas encapsulation in nanocarriers (such as liposomes, polymeric nanoparticles, dendrimers or inorganic–magnetic platforms) could prolong blood half-life, enhance tumor and brain accumulation, and reduce off-target toxicity (Bhadran et al., 2025; Radeva and Yoncheva, 2025). Nanoliposomes have become one of the most effective nanocarrier systems for improving DOX delivery across restrictive barriers such as the BBB (Izadiyan et al., 2025). Numerous studies show that liposomal formulations increase tumor uptake and decrease cardiotoxicity, as demonstrated by liposomal DOX and the FDA-approved product Doxil® (PEGylated liposomes, PLD), which validates their clinical utility in cancer therapy (Franco et al., 2018; Lee, 2019; Sercombe et al., 2015). Considering the poor pharmacokinetics of free DOX, nanoliposomes offer a rational strategy to enhance its therapeutic index and facilitate delivery into challenging tissues such as the brain (Cheng et al., 2025).

Surfactants such as Polysorbate 80 (Tween-80) are non-ionic polyoxyethylene sorbitan esters widely used as pharmaceutical excipients and as functional coatings on nanocarriers to promote transport across the BBB (Ravichandran et al., 2021). When nanoparticles are coated with Tween-80, they selectively adsorb plasma apolipoproteins especially ApoE and ApoB onto their surface, so that the resulting “protein corona” mimics endogenous lipoproteins and enables recognition by LDL-receptor family members on brain endothelial cells (Kreuter, 2013). Incorporating Tween-80 into polymeric nanoparticles has shown to enhance circulation time, increase interaction with brain capillary endothelium, and reduce the dose of free drug needed, which can translate into lower systemic toxicity (Kreuter, 2005). Classic *in vivo* studies demonstrated that polysorbate-80–coated poly(butyl cyanoacrylate) nanoparticles delivered drugs such as DOX, dalargin and loperamide efficiently to the brain achieving up to ∼60-fold higher brain levels compared with non-coated formulations providing strong experimental evidence that Tween-80–based surface modification is an effective strategy for enhancing drug delivery to the CNS (Gulyaev et al., 1999).

Despite advances in nanoparticulate drug-delivery systems, there remains an absence of comprehensive investigations examining Tween-80–coated nanoliposomes specifically loaded with DOX as a means to enhance drug transport across the intact BBB. As such, the present study aims to address this gap by formulating and comparing DOX-loaded nanoliposomes with varied lipid/Tween-80 compositions, characterizing their physicochemical properties and release behavior, and evaluating their potential to cross the BBB under basal *in vivo* conditions. No published studies have systematically evaluated how the ratio of Tween-80 to lipid, the lipid composition of the nanoliposome shell, and the interplay of these parameters affect key physicochemical attributes (size, zeta-potential, stability) together with drug-release kinetics. Furthermore, simultaneous assessment of formulation stability, surface charge, particle size distribution and *in-vitro*/*in-vivo* release profile of such DOX-loaded Tween-80 liposomes remains very limited, leaving a critical knowledge gap in optimizing these systems for brain-directed drug delivery.

## 2 Materials and methods

### 2.1 Materials

All chemicals and drugs used in this study were of analytical grade and obtained from reputable international suppliers. Methanol (99.9%), chloroform (99.8%), cholesterol (99%), ammonium sulfate ((NH₄)₂SO₄, 99%), histidine (99%), monopotassium phosphate (KH₂PO₄, 99%), ammonium molybdate tetrahydrate (99%), ascorbic acid (99%), hydrogen peroxide (30%), methyl thiazolyl tetrazolium bromide (MTT, >97%), trypan blue solution (0.4%), and DOX hydrochloride (>98%) were purchased from Sigma–Aldrich (St. Louis, MO, USA). Absolute ethanol (99.9%), isopropanol (99.9%), dextrose (99%), hydrochloric acid (1 M), concentrated sulfuric acid (98%, 36 N), dimethyl sulfoxide (DMSO, >99.9%), and histology-grade xylene were obtained from Merck (Darmstadt, Germany). Hydrogenated soy phosphatidylcholine (HSPC, 99%) was supplied by Lipoid (Ludwigshafen, Germany), while dipalmitoyl phosphatidylglycerol (DPPG, 99%) and distearoyl phosphatidylethanolamine-N-[methoxy(polyethylene glycol)] (mPEG-DSPE, 99%) were purchased from Avanti Polar Lipids (Alabaster, AL, USA). Tween® 80 (99%) was obtained from Merck (Germany). Phosphate-buffered saline (PBS), RPMI-1640 culture medium, fetal bovine serum (FBS), antibiotics, and trypsin–EDTA (0.25%) were purchased from Gibco (Thermo Fisher Scientific, USA). Injectable DOX hydrochloride (2 mg/mL) was obtained from Ebewe Pharma (Austria), and the commercial liposomal DOX formulation was supplied by Exir Nano Sina (Iran). For animal studies, ketamine and xylazine were purchased from Alfasan (Woerden, Netherlands).

### 2.2 Cell line

The murine GBM C6 cell line (ATCC® CCL-107™) and the normal murine fibroblast cell line NIH 3 T3 (ATCC® CRL-1658.2™) were obtained from the Pasteur Institute of Iran (Tehran, Iran) and maintained under standard cell culture conditions.

### 2.3 Experimental animals

Male BALB/c mice weighing approximately 20 g and aged 4–6 weeks (obtained from the Animal House of Mashhad University of Medical Sciences) were used in this study. Prior to experimental procedures, animals were acclimatized to the laboratory environment for 7 days. Mice were housed in standard polypropylene cages (*n* = 3–5 per cage) maintained under controlled environmental conditions (temperature: 22 ± 1 °C, relative humidity: 55 ± 5%, and a 12 h light/dark cycle) with access to a standard pellet diet and purified water. Animals were monitored daily for health status, body weight, and signs of distress; no animals were excluded due to unexpected illness or mortality during the study period. All animal experiments were conducted following the ethical parameters established by the Animal Management Rules of Research Advisory and Institutional Ethical Committees of Mashhad University of Medical Sciences (Ethical Approval Code: IR.MUMS.PHARMACY.REC.1398.046) and strictly adhered to the ARRIVE guidelines.

### 2.4 Preparation of DOX-loaded liposomes

DOX-loaded liposomes were prepared using the thin-film hydration method followed by extrusion, as previously described with minor modifications (Khabbazian et al., 2024). Lipid mixtures composed of HSPC, cholesterol, DPPG, and Tween® 80 at predefined molar ratios (Table 1) were dissolved in chloroform. A chloroform: methanol mixture (2:1, *v*/v) was used to obtain homogeneous lipid solutions. Organic solvents were removed under reduced pressure at 60 °C to form a thin lipid film, which was further dried to ensure complete solvent removal. The lipid films were hydrated with 0.25 M ammonium sulfate solution (pH 5.5) at 60 °C to form multilamellar vesicles. The resulting liposomal suspensions were subjected to mild sonication and subsequently extruded at 60–65 °C through polycarbonate membranes with decreasing pore sizes (200, 100, and 80 nm) to obtain nanosized liposomes with a narrow size distribution. To establish a transmembrane ion gradient for remote drug loading, the extruded liposomes were dialyzed against dextrose–histidine buffer (10 mM histidine, pH 5.6) at room temperature using sequential buffer exchanges. After dialysis, the liposomes were suitable for active loading of DOX and further physicochemical characterization. A PEGylated liposomal formulation analogous was prepared by replacing DPPG with mPEG-DSPE, while maintaining the total lipid concentration constant, as reported in previous studies on PEGylated liposomal DOX formulations.

**Table 1:** Molar composition of liposomal formulations.

Formulation	HSPC (mol%)	Cholesterol (mol%)	DPPG (mol%)	Tween® 80 (mol%)	mPEG-DSPE (mol%)
F1	56.75	38	5	0.25	–
F2	51.75	38	10	0.25	–
F3	51.5	38	10	0.5	–
F4	51	38	10	1	–
F5	49.5	38	10	2.5	–
F6	47	38	10	5	–
F7 (PEGylated)	57	38	–	–	5

HSPC: Hydrogenated Soy Phosphatidylcholine, DPPG: Dipalmitoyl phosphatidylglycerol, mPEG-DSPE: methoxy Poly (ethylene glycol)-Distearoylphosphatidylethanolamine.

### 2.5 Phosphate assay

The phospholipid contents of the liposomal formulations was quantified using a phosphate colorimetric assay based on the Bartlett method (Bartlett, 1959), which was selected over the Stewart method for its higher sensitivity (Itoh et al., 1986; Stewart, 1980). This assay is based on acid digestion of phospholipids, resulting in the release of inorganic phosphate, which reacts with ammonium molybdate to form phosphomolybdic acid. Subsequent reduction with ascorbic acid yields a blue-colored complex with a maximum absorbance at 800–830 nm. To avoid interference from phosphate-containing buffers, the assay was applied only to liposomal formulations prepared in phosphate-free media. Accurate temperature control during digestion was maintained, as incomplete phosphate release occurs below 180 °C, while temperatures above 200 °C lead to acid evaporation and reduced assay accuracy. Briefly, 10 μL of each liposomal formulation was mixed with 40 μL of distilled water in glass tubes. For standard curve preparation, different volumes of a 0.65 mM potassium dihydrogen phosphate (KH₂PO₄) solution were used, and a blank sample without phosphate was included. Subsequently, 400 μL of 10 N sulfuric acid was added to each tube, and samples were digested at 190–200 °C for 1 h using a heating block. After cooling to room temperature for 15 min, 100 μL of freshly prepared 10% H_2_O_2_ was added, followed by further heating at 200 °C for 10 min. The samples were then incubated in a boiling water bath for 10 min to remove residual carbon as CO₂. After cooling, 1 mL of ammonium molybdate reagent and 500 μL of freshly prepared ascorbic acid solution (0.1 g/mL) were sequentially added to each tube. The mixtures were vortexed briefly and incubated in a boiling water bath for 20 min to allow color development. The tubes were then rapidly cooled on ice, and absorbance was measured at 800 nm using a UV–Vis spectrophotometer (UV-1800, Shimadzu Corporation, Kyoto, Japan). All standard and sample measurements were performed in independent triplicates (*n* = 3). A phosphate calibration curve was constructed using the KH_2_PO_4_ standards, and the phospholipid content of the liposomal samples was calculated from the linear regression equation of the standard curve and reported as mean ± standard deviation (SD).

### 2.6 Drug loading

DOX was actively loaded into the preformed liposomal formulations using the ammonium sulfate gradient (remote loading) method, as previously described (Hosseinikhah et al., 2025). Briefly, liposomal suspensions obtained after buffer exchange were equilibrated at 37 °C, while the DOX solution was preheated at 68 °C for 1 h to facilitate transmembrane drug diffusion. The amount of DOX added to each formulation was calculated based on the phospholipid-to-drug ratio determined from the measured phospholipid content. Aliquots of 1 mL of each liposomal suspension were incubated with the calculated amount of DOX under gentle mixing to allow active drug encapsulation.

### 2.7 Fluorometric determination of DOX

Encapsulated DOX was quantified by spectrofluorimetry at excitation and emission wavelengths of 450 and 585 nm, respectively. Calibration curves were prepared using serial dilutions of injectable DOX (0–0.04 mg/mL) in acidified isopropyl alcohol (90% isopropanol, 0.075 M HCl) to ensure complete liposome disruption. Fluorescence intensity was recorded immediately, and DOX concentration was determined from the linear regression of the standard curve (Hosseinikhah et al., 2025).

### 2.8 Drug release evaluation

The release behavior of DOX from liposomal formulations was evaluated under different pH conditions (pH 7.4, 6.5, and 5.5) to simulate physiological, mildly acidic tumor, and acidic endosomal environments, respectively. Briefly, 0.5 mL of each DOX-loaded liposomal formulation was placed into dialysis bags (molecular weight cutoff: 12–14 kDa) and immersed in 50 mL of the corresponding release medium, including phosphate buffer (pH 7.4), dextrose–histidine buffer (pH 6.5), and dextrose–succinate buffer (pH 5.5). The system was maintained under gentle agitation, and at predetermined time intervals (0, 15, 30, and 60 min, followed by 2, 4, 6, 8, 12, and 24 h), 2 mL of the release medium was withdrawn and immediately replaced with an equal volume of fresh buffer to maintain sink conditions. The collected samples were analyzed for DOX content, and cumulative drug release was calculated according to a previously reported equation accounting for sample replacement (Hosseinikhah et al., 2025).

### 2.9 Physicochemical characterization of nanoliposomes

#### 2.9.1 Particle size, polydispersity index (PDI), and zeta potential

Particle size, PDI, and zeta potential of liposomal formulations before and after DOX loading were measured using a Zetasizer Nano ZS (Malvern Instruments, Worcestershire, UK). Particle size and PDI were determined by dynamic light scattering (DLS) at 25 °C and a scattering angle of 173° after appropriate dilution with dextrose-histidine buffer, while zeta potential was measured by laser Doppler electrophoresis at 25 °C following suitable sample preparation.

#### 2.9.2 Stability evaluation

The long-term physical stability of the selected liposomal formulation (F2) was evaluated by monitoring their physicochemical properties over a 3-month storage period. The liposomal samples were stored at 4 °C in the dark. At the end of this period, the samples were re-analyzed for mean particle size, PDI, and zeta potential using the Zetasizer Nano ZS as previously described. Any significant change in these parameters would be indicative of physical instability or particle aggregation.

#### 2.9.3 Transmission electron microscopy (TEM)

The morphology and structural characteristics of the liposomal formulations were evaluated by TEM. Freshly prepared liposomal samples were placed on carbon-coated copper grids, negatively stained with 2% (*w*/*v*) uranyl acetate, and air-dried at room temperature. Imaging was performed at an accelerating voltage of 80 kV using a Tecnai G2 Spirit TEM (FEI Company, Thermo Fisher Scientific, USA). Particle size distribution was quantified by measuring the hydrodynamic/geometric diameter of at least 125 randomly selected individual vesicles from TEM micrographs using ImageJ software. The resulting size distribution data were fitted to a lognormal distribution model, and the goodness-of-fit was evaluated using the adjusted coefficient of determination (Adj.R^2^).

### 2.10 MTT cell viability assay

The cytotoxicity of DOX-loaded liposomal formulations was evaluated using the MTT assay on murine GBM C6 cells. Cells were seeded in 96-well plates at a density of 5 × 10^3^ cells per well and incubated for 24 h at 37 °C in a humidified atmosphere containing 5% CO_2_. Cells were then treated with serial dilutions of liposomal formulations and free DOX at equivalent DOX concentrations and incubated for 48 h. Subsequently, the culture medium was replaced with MTT solution and incubated for 3.5–4 h to allow the formation of formazan crystals. The crystals were dissolved using DMSO, and absorbance was measured at 570 nm using a microplate reader. Furthermore, to evaluate biosafety and potential non-specific toxicity, the assay was similarly performed on NIH 3 T3 under identical incubation and treatment conditions. To evaluate carrier-related cytotoxicity and exclude non-specific toxicity of the delivery vehicle, empty nanoliposomal formulations (blanks of F2, F3, and F4 without DOX) were tested on normal NIH/3 T3.2 cells across equivalent lipid concentrations ranging from 0.31 to 10 μg/mL using the same MTT assay protocol.

### 2.11 *In vivo* Biodistribution study

The tissue distribution of Animals were randomly allocated into treatment groups (*n* = 3 per group/time point) using a computer-generated randomization sequence. DOX following intravenous administration was evaluated in healthy male BALB/c mice. Mice were injected *via* the tail vein with DOX-loaded nanoliposomes (F2, F3, F4, PDL), free DOX, or PBS as a control at a dose of 15 mg/kg DOX equivalent. At predetermined time points post-injection (2, 4, 8, 24, and 48 h), blood samples were collected *via* retro-orbital bleeding under light anesthesia. Prior to tissue collection, animals were deeply anesthetized *via* intraperitoneal injection of ketamine (100 mg/kg) and xylazine (10 mg/kg) and transcardially perfused with ice-cold phosphate-buffered saline (PBS, pH 7.4) through the left ventricle until systemic circulation was thoroughly flushed and tissues were blanched ensuring complete clearance of residual intravascular blood. Subsequently, animals were euthanized by cervical dislocation and major organs, including brain, heart, liver, spleen, kidneys, and lungs, were harvested. DOX concentrations in plasma and tissue samples were quantified, and biodistribution profiles were compared among the different formulations to assess tissue accumulation, with particular emphasis on brain uptake (Hosseinikhah et al., 2025).

### 2.12 Acute tissue toxicity and histopathological evaluation

Acute tissue toxicity of DOX-loaded nanoliposomal formulations was evaluated in BALB/c mice. Animals were randomly assigned into experimental groups (*n* = 5 per group) and intravenously administered *via* the tail vein with DOX-loaded nanoliposomes, free DOX, or PBS as control on days 1, 8, and 15 at an equivalent DOX dose of 6 mg/kg. Animal survival and body weight changes were monitored until day 21. On day 28, animals were humanely euthanized, and major organs, including brain, heart, liver, spleen, lungs, and kidneys, were harvested, fixed in 10% neutral-buffered formalin, embedded in paraffin, sectioned (≈5 μm), and stained with hematoxylin and eosin (H&E). Histopathological evaluation and semi-quantitative scoring of tissue damage were performed under light microscopy by a pathologist in a blinded manner to avoid observer bias (Rahchamandi et al., 2024).

### 2.13 Statistical analysis

All data are presented as mean ± SD, with the individual animal serving as the experimental unit for *in vivo* analyses. TEM images were analyzed for particle size distribution using ImageJ software (National Institutes of Health, Bethesda, MD, USA), and additional data plotting was performed using Origin software (OriginLab Corporation, Northampton, MA, USA). Statistical analysis for *in vitro* assays was performed using one-way analysis of variance (ANOVA), followed by Dunnett's post-hoc for multiple comparisons. For *in vivo* animal studies, given the sample size (*n* = 3 per group) and potential data variability, normality cannot be assumed; therefore, non-parametric analysis was applied using the Kruskal–Wallis test followed by Dunn's post-hoc test for multiple-comparison correction. A *p*-value <0.05 was considered statistically significant. For parametric evaluations, including semi-quantitative histopathological scoring, differences among groups were analyzed using two-way ANOVA followed by Dunnett's post-hoc test for multiple comparisons against the control group. Statistical analyses and primary graphical representations were carried out using GraphPad Prism software (version 9.5.1, GraphPad Software Inc., San Diego, CA, USA). All experiments were conducted in three independent replicates.

## 3 Results

### 3.1 Post-dialysis DLS characterization of liposomal formulations

**Fig. 1:**
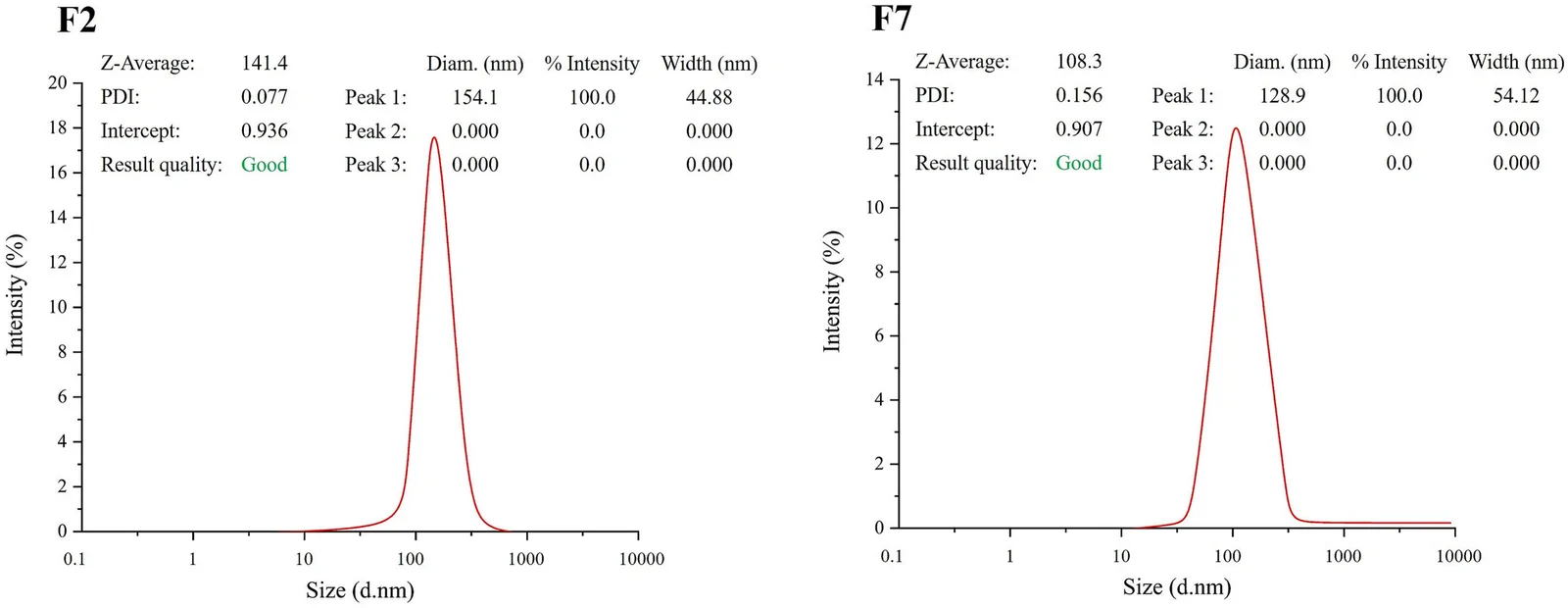
Representative DLS intensity size distributions of dialyzed free liposomal formulations: the lead Tween 80-containing formulation F2 and the benchmark PEGylated formulation F7 after dialysis against dextrose-histidine buffer.

Post-dialysis DLS characterization confirmed that all seven liposomal formulations remained within the nanoscale range after dialysis against dextrose-histidine buffer, with mean particle sizes ranging from 108.3 to 141.4 nm (Fig. 1 and Fig. S1) (Supplementary Information). The smallest particles were observed for F7 (108.3 nm) and F3 (112.8 nm), whereas the lead formulation F2 exhibited a mean diameter of 141.4 nm. Size distributions were narrow for most formulations, with PDI values ranging from 0.051 to 0.156, while F6 displayed higher dispersity (PDI 0.277) with a minor oversized population. All formulations carried a negative surface charge, with zeta potentials ranging from −23.8 to −37.7 mV for F7 and F5, respectively.

### 3.2 TEM characterization of free liposomal nanoparticles

**Fig. 2:**
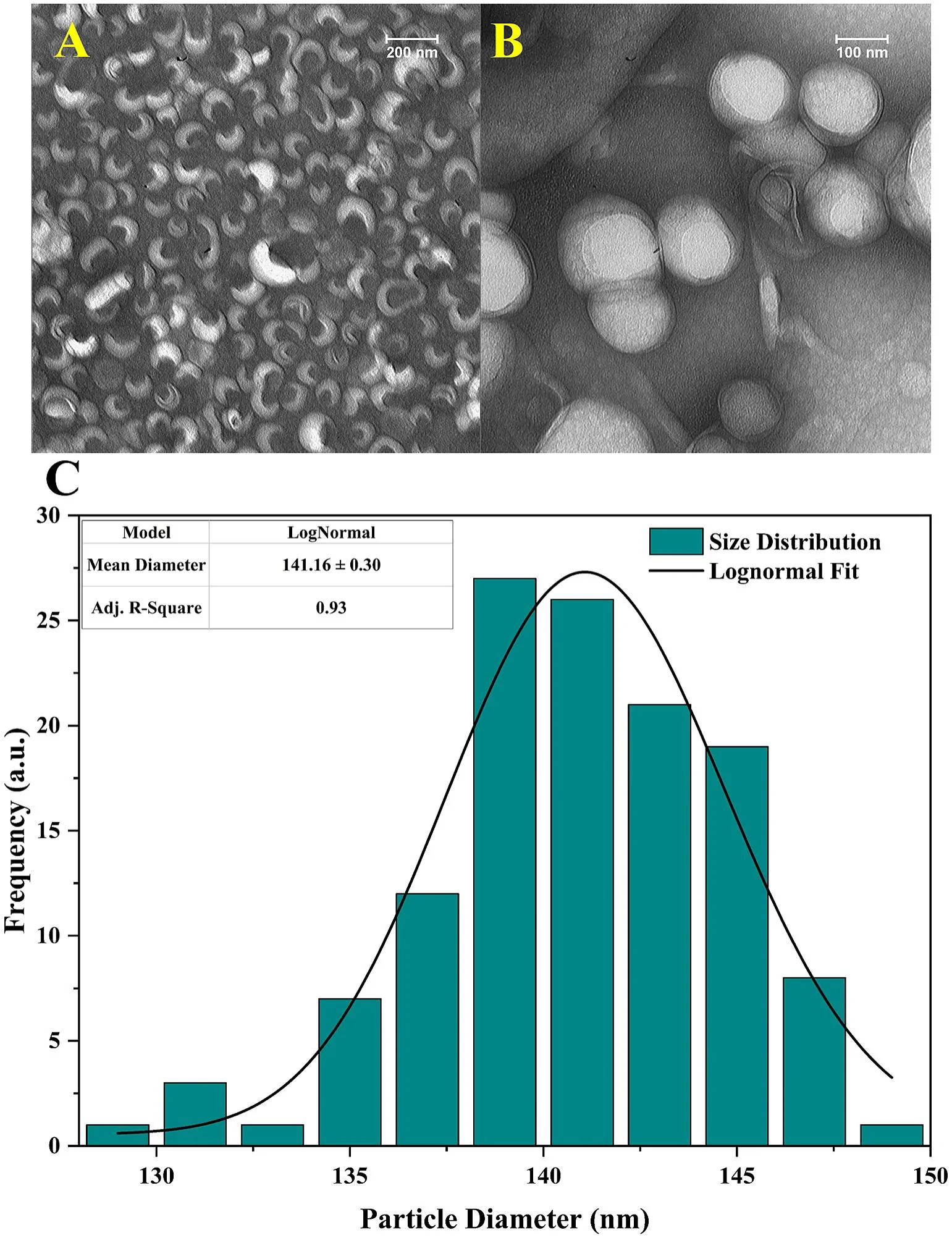
TEM images of liposomal nanoparticles (F2) at low (A) and high (B) magnification, showing nanosized vesicles with well-defined bilayer structures. (C) Particle size distribution histogram obtained from TEM image analysis of 125 randomly selected vesicles (n = 125), overlaid with a lognormal distribution fit. The goodness-of-fit (Adj. R^2^ = 0.93) and mean diameter are presented in the inset table.

As shown in Fig. 2, TEM images demonstrated the formation of nanosized liposomal vesicles (F2) with predominantly spherical morphology and no evident aggregation. High-magnification images confirmed the presence of well-defined bilayer structures. Quantitative size analysis of 125 randomly selected vesicles (*n* = 125) from TEM micrographs yielded a mean particle diameter of 141.16 ± 0.30 nm. The particle size distribution was well-described by a lognormal fit with an adjusted R^2^ (Adj.R^2^) of 0.93, demonstrating a highly reliable goodness-of-fit and confirming a narrow, monodisperse size distribution in good agreement with the DLS measurements.

### 3.3 Phosphate (Bartlett) assay

Phospholipid contents of the liposomal formulations were quantified using the Bartlett colorimetric assay. A linear calibration curve was obtained using KH_2_PO_4_ standards over the concentration range of 30.55 to 152.57 nmol/tube (R^2^ = 0.996). The final total phospholipid contents for formulations F1 through F7 (after sample volume normalization) were determined to be 38.5 ± 0.7, 36.6 ± 0.5, 36.0 ± 0.4, 30.6 ± 0.9, 43.2 ± 0.2, 24.0 ± 1.8, and 33.0 ± 1.2 nmol, respectively.

### 3.4 Characterization of DOX-loaded nanoliposomes

**Fig. 3:**
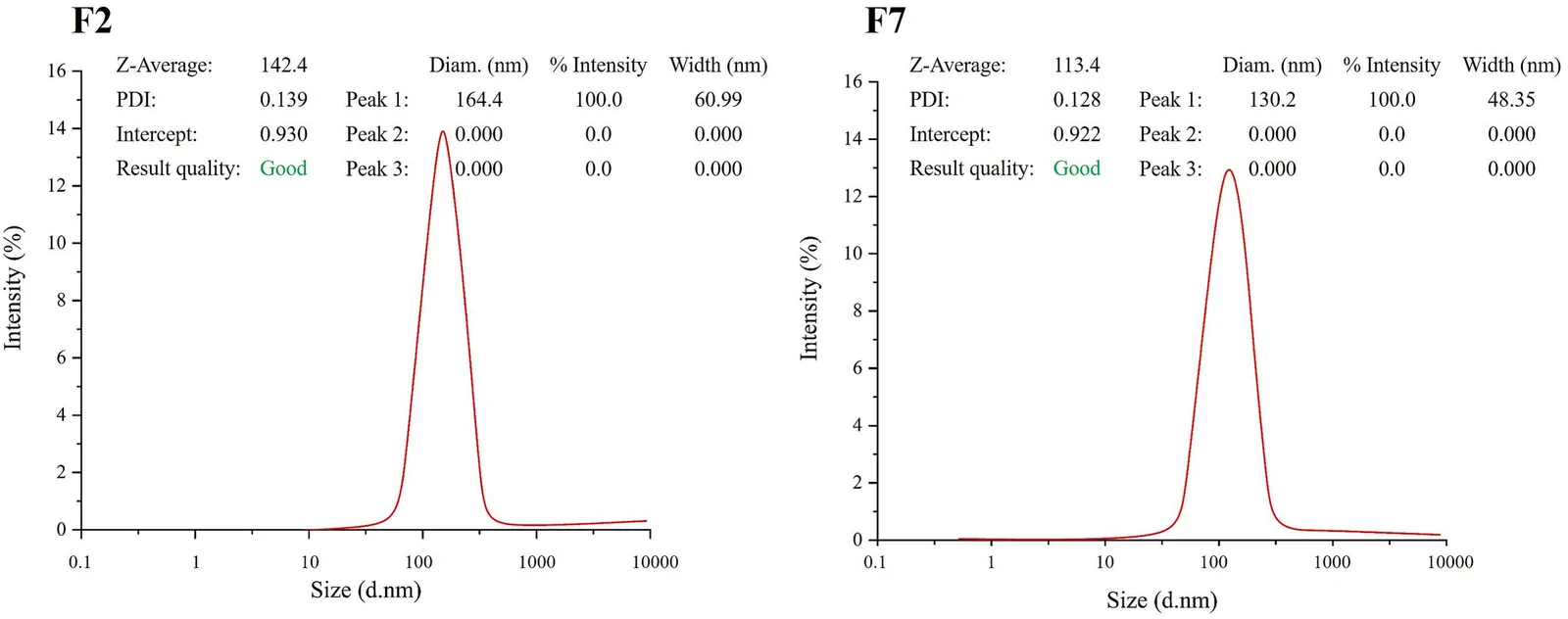
Representative DLS intensity size distribution profiles of DOX-loaded nanoliposomes: the lead Tween 80-containing formulation F2 and the benchmark PEGylated formulation F7 after active remote drug loading.

Following active remote loading of DOX, DOX-loaded nanoliposomes exhibited mean particle sizes ranging from 113.4 to 275.9 nm, with PDI values remaining below 0.36 across all formulations (Fig. 3 and Fig. S2). Formulations F1, F2, F3, and F7 maintained their structural integrity and monodisperse size distribution after drug loading, whereas F4, F5, and F6 displayed a marked increase in both mean particle size and PDI, indicating particle aggregation and membrane instability. Zeta potential values remained negative across all formulations, ranging from −15.9 to −33.9 mV, consistent with acceptable colloidal stability. To highlight the primary formulation and its benchmark control, representative DLS intensity profiles after DOX loading.

As shown in Table 2, formulations F1, F2, F3, and F7 maintained their structural integrity and size stability after DOX loading. In contrast, F4, F5, and F6 exhibited a significant increase in both mean particle size and PDI upon drug encapsulation, indicating physical instability and particle aggregation. This numerical data strongly supports our decision to exclude these unstable formulations from further *in vivo* investigations. The encapsulation efficiency of the nanoliposomal formulations is also presented in Table 2. Encapsulation efficiency varied across formulations, with values between 61.76% and 115.49%, indicating formulation-dependent differences in drug loading.

**Table 2:** Physicochemical properties and size stability of different nanoliposomal formulations before and after doxorubicin loading.

Formulation	Condition	Mean Particle Size (nm)	PDI	Zeta Potential (mV)	Encapsulation efficiency (%)
F1	Before loading	130.2	0.101	−26.9	–
	After loading	129.5	0.096	−22.6	105.26
F2	Before loading	141.4	0.077	−33.0	–
	After loading	142.4	0.139	−15.9	82.42
F3	Before loading	112.8	0.051	−36.7	–
	After loading	116.0	0.090	−32.1	115.49
F4	Before loading	113.7	0.149	−35.2	–
	After loading	161.0	0.255	−17.1	108.75
F5	Before loading	120.8	0.156	−37.7	–
	After loading	275.9	0.309	−33.9	61.76
F6	Before loading	127.8	0.277	−27.5	–
	After loading	269.6	0.356	−17.2	81.68
F7	Before loading	108.3	0.156	−23.8	–
	After loading	113.4	0.128	−23.7	96.55

### 3.5 Long-term storage stability

The physical stability of F2 during storage was investigated to ensure the integrity of the liposomes over time. The results summarized in Table 3 indicate that there were no significant changes in the particle size, PDI, or zeta potential of the formulations after 3 months of storage at 4 °C. These findings suggest that the liposomal formulations are stable and maintain their colloidal properties under these storage conditions.

**Table 3:** Long-term physical stability of F2 liposomal formulations stored at 4 °C over a 3-month period.

Formulation	Storage time	Mean particle size (nm)	PDI	Zeta potential (mV)
F2	0 days (Initial)	142.4	0.139	−15.9
	3 months	139.2	0.049	−30.3

### 3.6 *In vitro* DOX release profiles

**Fig. 4:**
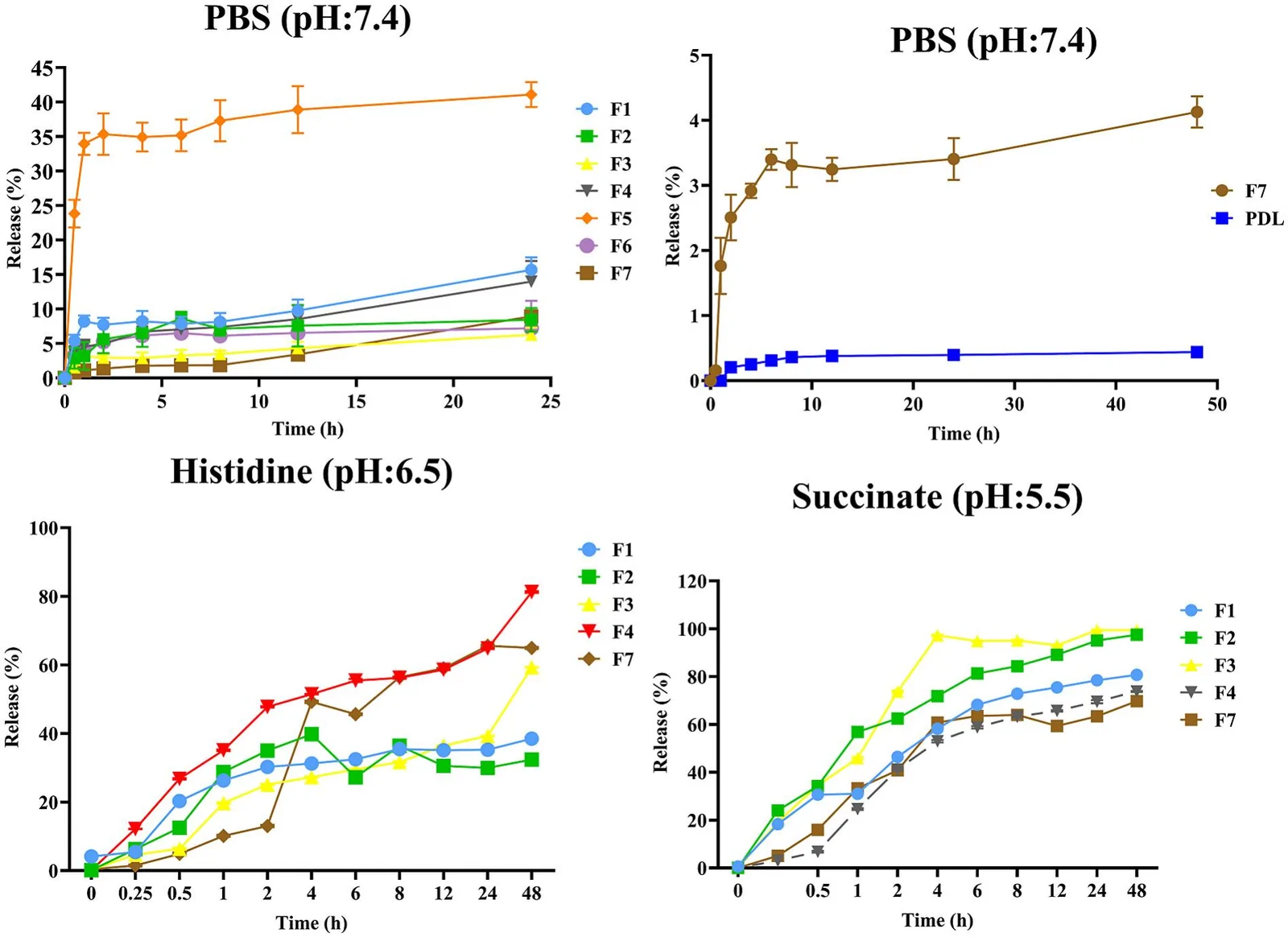
*In vitro* release profiles of DOX from liposomal formulations under different pH conditions: PBS (pH 7.4), comparative release of formulation F7 and commercial PDL in PBS (pH 7.4), histidine buffer (pH 6.5), and succinate buffer (pH 5.5). Data are presented as mean ± SD (*n* = 3). In some cases, error bars are smaller than the plotted symbols and are therefore covered by the data points.

As shown in Fig. 4, DOX release from all formulations in PBS (pH 7.4) was limited and time-dependent. Most formulations released less than 15% of the drug after 24 h, except for formulation F5, which showed the highest release of approximately 40% (*P* < 0.001). Formulations F3 and F7 exhibited the lowest release, remaining below 10% throughout the study period. In succinate buffer (pH 5.5), a pronounced increase in drug release was observed for all formulations. An initial rapid release phase occurred within the first hours, followed by a plateau after 8–12 h. Formulation F3 reached nearly complete drug release within 12 h, while formulations F1and F2 achieved high release levels of approximately 80–90%. In contrast, formulations F4 and F7 showed lower cumulative release, remaining around 60–70%. In Release profiles in histidine buffer, Formulation-dependent differences were evident, with formulation F4 exhibiting the highest cumulative release, reaching approximately 85% after 48 h (P < 0.001). Formulation F7 followed with about 65% release, whereas formulations F1, F2, and F3 displayed lower release levels in the range of 30–50% (*P* < 0.01).

### 3.7 *In vitro* cytotoxicity

**Fig. 5:**
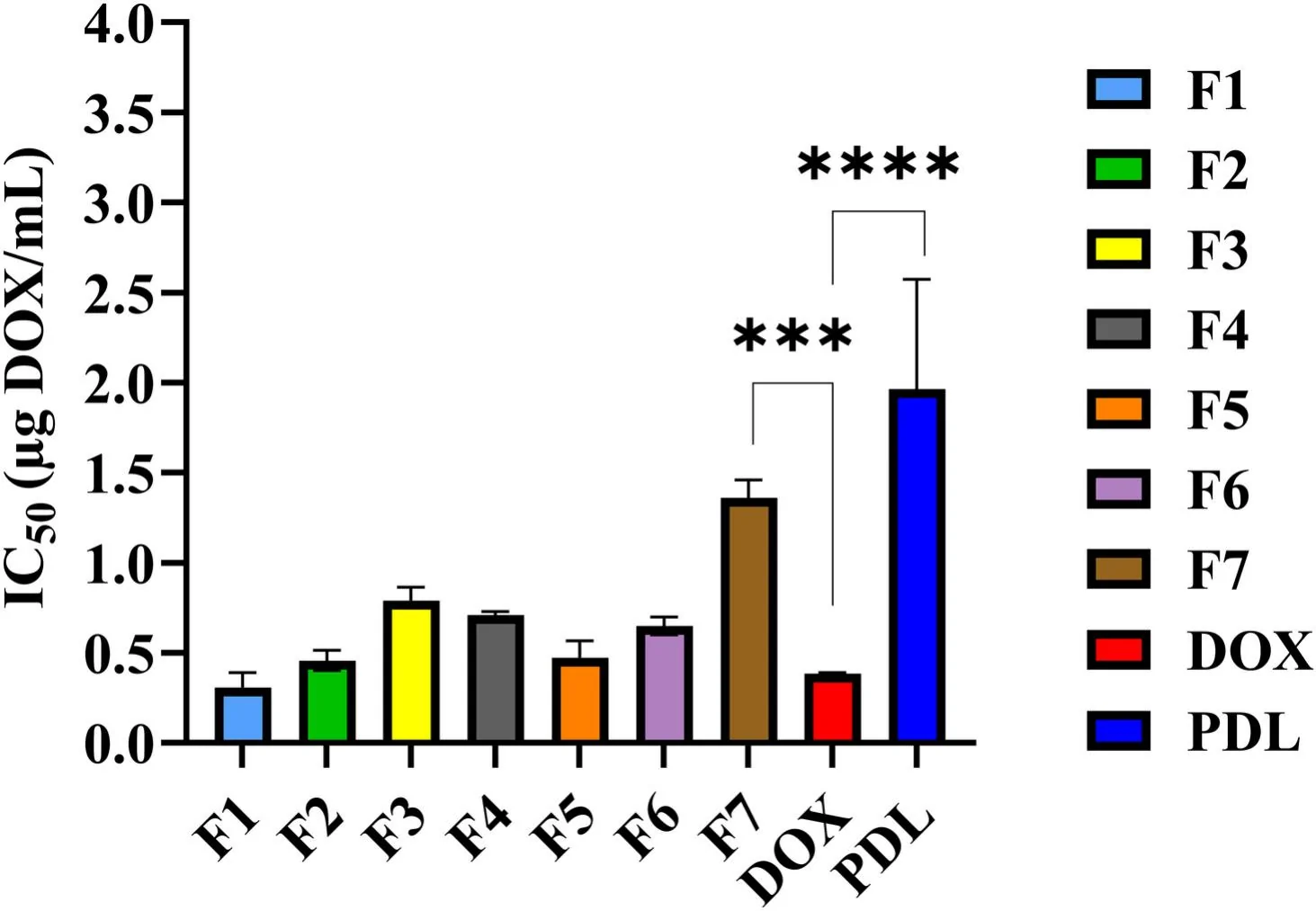
IC_50_ values of DOX-loaded nanoliposomal formulations (F1–F7), free DOX, and commercial PDL determined by the MTT assay in C6 glioblastoma cells. All IC_50_ values are expressed based on equivalent doxorubicin concentration (μg DOX/mL). Data represent mean ± SD (*n* = 3). ***p < 0.001 and ****p < 0.0001 compared to free DOX.

As shown in Fig. 5, the cytotoxic effects of DOX-loaded nanoliposomal formulations were assessed by the MTT assay, and IC_50_ values were determined to evaluate anticancer potency. All treatment concentrations and calculated IC_50_ values specifically refer to the equivalent concentration of DOX concentration, rather than total lipid or total nanoliposomal formulation mass, ensuring a direct and standardized comparison with free DOX. The IC_50_ values for the nanoliposomal formulations ranged from 0.308 to 1.360 μg/mL of DOX. Free DOX exhibited an IC_50_ of 0.383 μg/mL of DOX, indicating its baseline cytotoxicity under the tested conditions. Among the formulations tested, F7 demonstrated an IC_50_ value of 1.360 μg/mL of DOX, which was significantly higher than that of free DOX (*p* = 0.0002), reflecting a notable decrease in cytotoxic activity. Similarly, the PDL group showed an even greater increase in IC_50_, with a value of 1.965 μg/mL of DOX, also significantly higher than free DOX (*p* < 0.0001), suggesting reduced efficacy in cellular toxicity. In contrast, the IC_50_ values of the other nanoliposomal formulations (F1 through F6) did not differ significantly from free DOX (*p* > 0.05), indicating comparable cytotoxic effects.

**Fig. 6:**
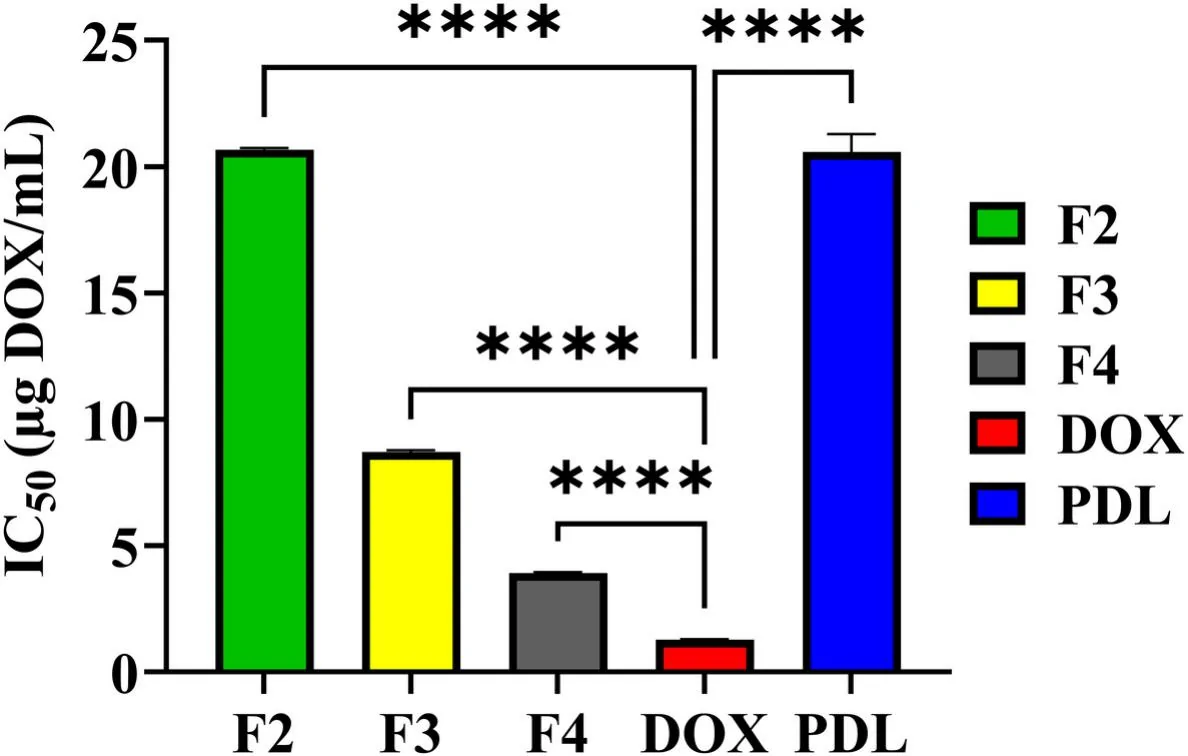
Cytotoxicity evaluation (IC_50_ values) of DOX, PDL, and nanoliposomal formulations (F2, F3, F4) in NIH 3 T3 cells determined by the MTT assay after 48 h incubation. All IC_50_ values are expressed based on equivalent DOX concentration (μg DOX/mL). Data represent Mean ± SD (n = 3). ****p < 0.0001 indicates a statistically significant difference compared to free DOX.

Furthermore, as illustrated in Fig. 6, evaluation on NIH 3 T3 cells demonstrated that DOX is highly toxic IC_50_ = 1.29 μg/mL, whereas the lead formulation F2 significantly protected healthy cells by exhibiting a markedly higher IC_50_ of 20.67 μg/mL (*p* < 0.0001 *vs.* DOX). In addition, to evaluate carrier-related cytotoxicity, empty nanoliposoms (blank formulations F2, F3, and F4) were tested on normal NIH 3 T3 cells across equivalent lipid concentrations ranging from 0.31 to 10 μg/mL. As presented in Fig. S3, all blank formulations maintained high cell viability above 90% across all tested concentrations, confirming the excellent biocompatibility and safety of the nanocarrier system without intrinsic toxicity.

### 3.8 *In vivo* biodistribution of DOX

**Fig. 7:**
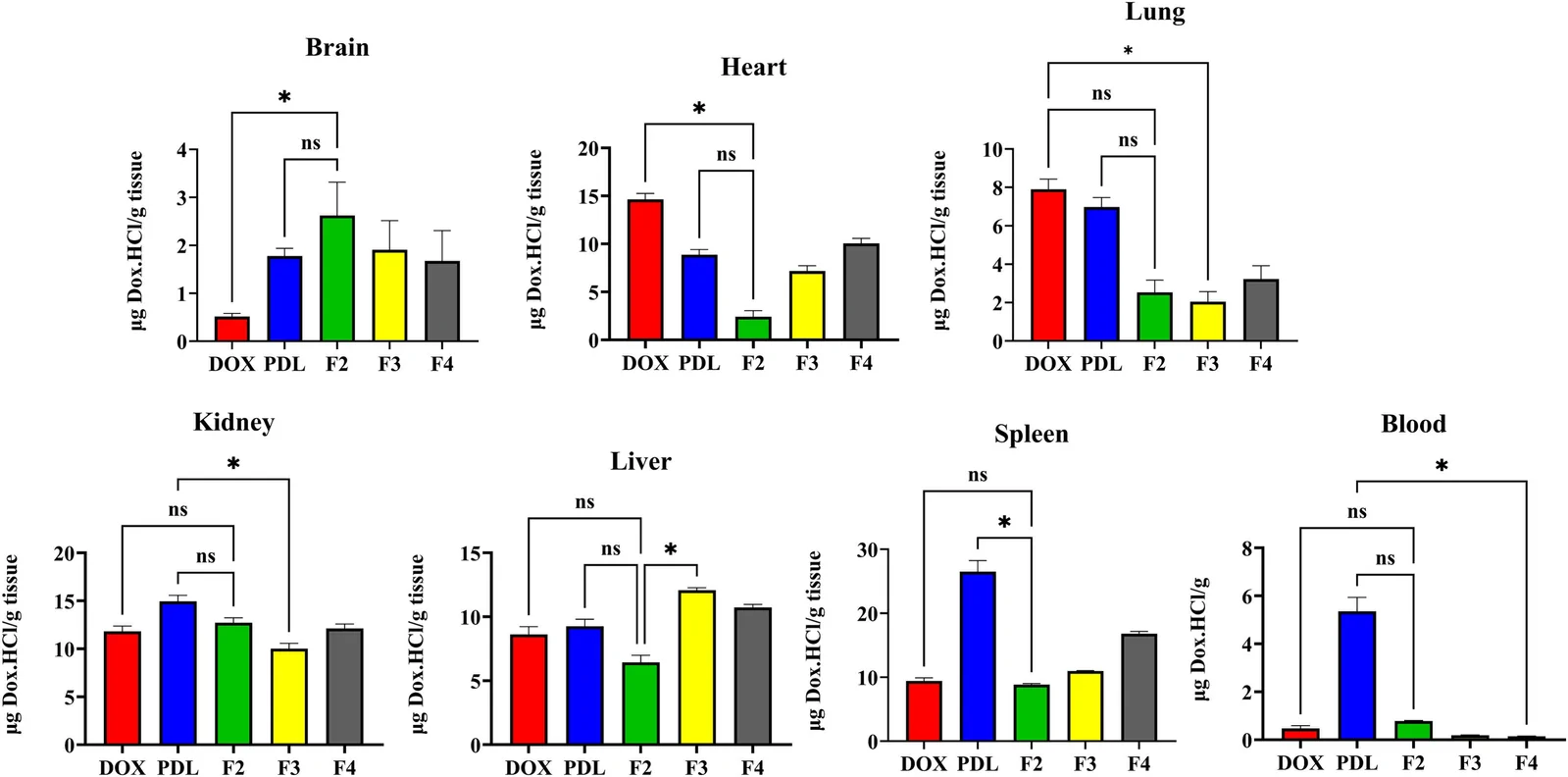
Tissue distribution of DOX at 24 h post-injection. Mean concentration of DOX (μg DOX·HCl/g tissue) in brain, heart, lung, kidney, liver, spleen, and blood of BALB/c mice 24 h after intravenous administration of free DOX, PDL, and nanoliposomal formulations (F2–F4) at a dose of 15 mg/kg. (Data are presented as mean ± SD, *n* = 3 animals per group. *p < 0.05 indicates statistical significance determined by Kruskal–Wallis test followed by Dunn's post-hoc test. For clarity, only statistically significant differences (p < 0.05) and selected key non-significant comparisons (ns) are indicated on the graphs; other non-essential non-significant comparisons are omitted.

**Fig. 8:**
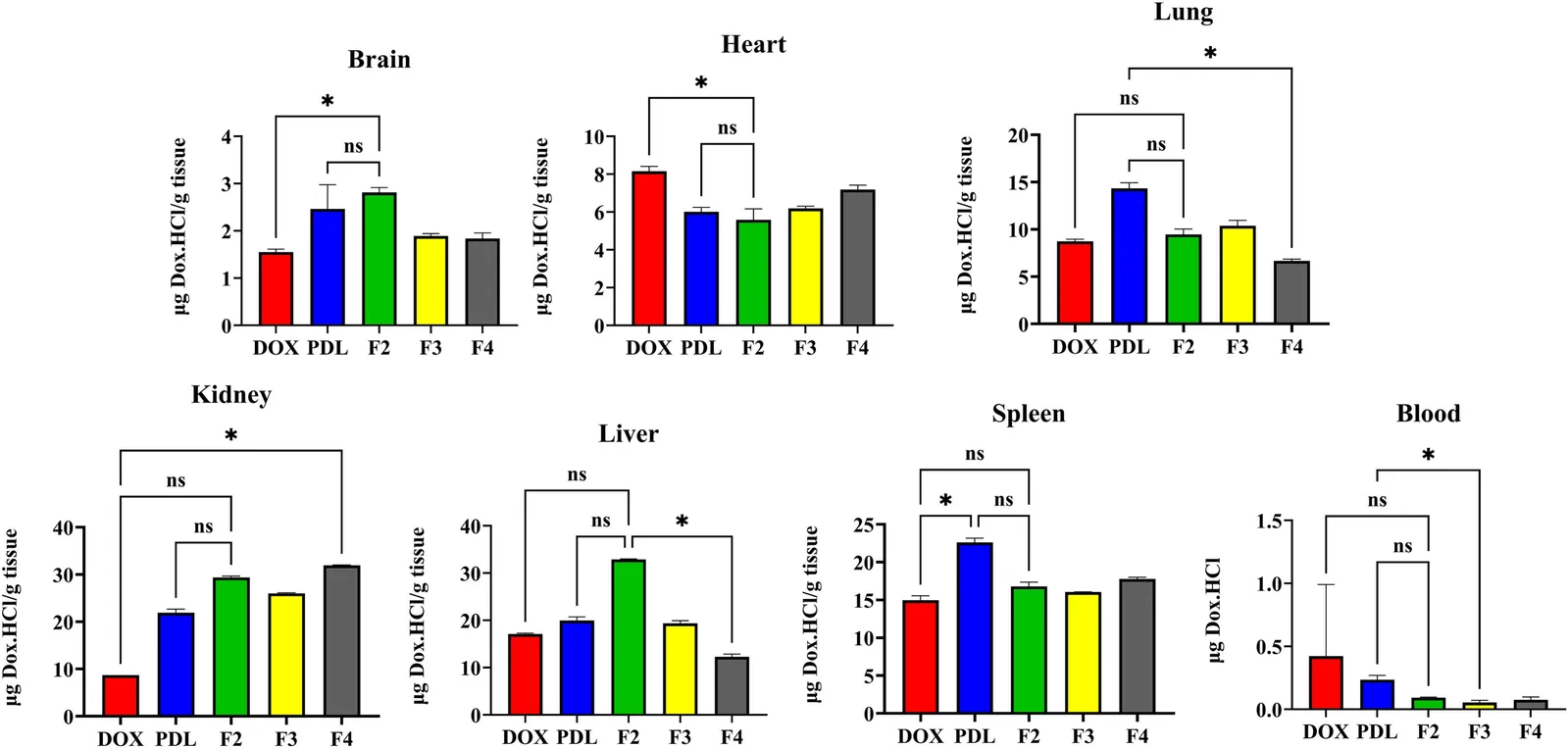
Tissue distribution of DOX at 48 h post-injection. Mean concentration of DOX (μg DOX·HCl/g tissue) in brain, heart, lung, kidney, liver, spleen, and blood of BALB/c mice 48 h after intravenous administration of free DOX, PDL, and nanoliposomal formulations (F2–F4). (Data are presented as mean ± SD (n = 3). *p < 0.05 indicates statistical significance. For clarity, only statistically significant differences (p < 0.05) and selected key non-significant comparisons (ns) are indicated on the graphs; other non-essential non-significant comparisons are omitted.

The biodistribution of DOX was evaluated in major organs of healthy BALB/c mice (n = 3 animals per group per time point) at 24 and 48 h post-intravenous injection (15 mg/kg equivalent DOX dose; Fig. 7, Fig. 8). All animals survived the procedures and completed the study time points without exclusion. Overall, tissue accumulation patterns were strongly organ- and time-dependent. At 24 h post-injection, DOX accumulation in brain tissue differed among the tested groups. Formulation F2 showed the highest brain accumulation (2.61 ± 0.69 μg/g tissue), exhibiting a statistically significant increase compared with DOX (*p* < 0.05). However, differences between F2 and PDL or other formulations (F3 and F4) were not statistically significant (*p* > 0.05). At 48 h post-injection, F2 maintained elevated brain accumulation (2.78 ± 0.12 μg/g tissue) and demonstrated a statistically significant increase compared with free DOX (*p* < 0.05).

In heart tissue, free DOX displayed the highest accumulation at 24 h. Among the tested formulations, F2 demonstrated a pronounced and statistically significant reduction in cardiac uptake compared to free DOX (*p* < 0.05), achieving the lowest accumulation in heart tissue (2.29 ± 0.77 μg/g tissue). At 48 h, free DOX continued to show significantly higher accumulation compared to F2 (*p* < 0.05), while differences between F2 and PDL remained non-significant. In lung tissue at 24 h, F3 displayed a statistically significant reduction in accumulation compared to free DOX (p < 0.05). At 48 h, F4 showed a statistically significant decrease in lung uptake compared to free DOX (p < 0.05), while accumulation levels among DOX, PDL, and F2 remained comparable (p > 0.05). Kidney tissue showed substantial DOX accumulation over time. At 24 h, F3 demonstrated a statistically significant decrease in renal uptake compared to free DOX (p < 0.05). At 48 h, F4 exhibited a statistically significant increase in renal accumulation compared to free DOX (p < 0.05).

Liver and spleen exhibited high DOX uptake across formulations, consistent with reticuloendothelial system clearance. In the liver at 24 h, F3 showed a statistically significant increase in uptake compared to F2 (*p* < 0.05). At 48 h, F2 exhibited a pronounced increase in hepatic accumulation, showing a statistically significant higher level compared to F4 (*p* < 0.05). In the spleen at 24 h, PDL exhibited a statistically significant higher accumulation compared to F2 (p < 0.05). At 48 h, PDL maintained a statistically significant higher accumulation compared to free DOX (p < 0.05). In blood at 24 h, F4 demonstrated a statistically significant reduction in circulating drug levels compared to free DOX (p < 0.05). At 48 h, circulating levels decreased across all groups; F4 maintained a statistically significant lower blood level compared to free DOX (*p* < 0.05).

The overall biodistribution profile reveals a therapeutic advantage for the developed nanoliposomes (F2–F4). These formulations exhibited reduced accumulation in off-target tissues such as the heart and lungs, particularly at 24 h. Concurrently, F2 consistently demonstrated sustained brain accumulation across the intact BBB at both 24 and 48 h, suggesting its potential for brain-directed delivery in baseline animal models while minimizing the risk of systemic side effects and normal-tissue toxicity.

### 3.9 Histopathological analysis

**Fig. 9:**
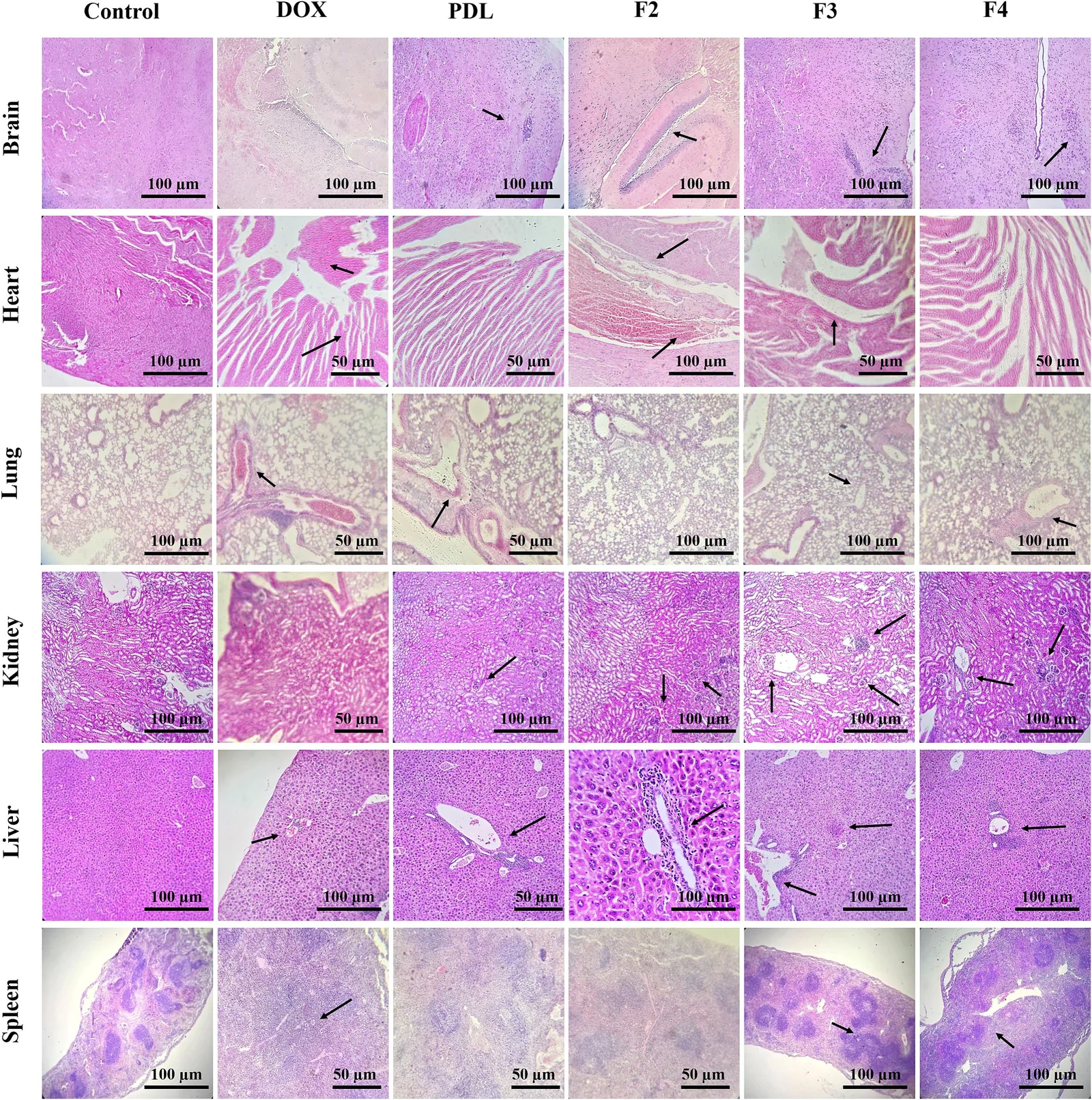
Representative H&*E*-stained histopathological sections of organ tissues. Organs were harvested from male BALB/c mice (*n* = 5 animals per group) following intravenous administration of PBS (Control), free DOX, PDL, and nanoliposomal formulations (F2, F3, F4) at an equivalent DOX dose of 6 mg/kg on days 1, 8, and 15. Black arrows highlight key pathological features across organs: Brain: Focal perivascular inflammation (PDL, F2, F3, F4). Heart: Interstitial edema and myofiber disorganization (DOX), and mild interstitial widening (F2, F3). Lung: Moderate vascular congestion (DOX), focal peribronchial inflammation (PDL), and inter-alveolar septal thickening (F3, F4). Kidney: Focal tubular degeneration and vacuolization (PDL, F2, F4), and severe degeneration with focal necrosis (F3). Liver: Periportal degeneration (DOX), subtle inflammation (PDL), and marked periportal inflammation with hepatocellular degeneration (F2, F3). Spleen: Lymphoid depletion and red pulp congestion (DOX), and prominent lymphoid follicle hyperplasia (F3, F4). All histopathological evaluations were performed by an experienced pathologist blinded to group allocations. (Scale bars represent 100 μm for images taken at 100× magnification and 50 μm for images taken at 400× magnification). (For interpretation of the references to color in this figure legend, the reader is referred to the web version of this article.)

Fig. 9 illustrates the representative histopathological alterations observed in organs harvested from BALB/c mice (*n* = 5 animals per group) following intravenous administration of different DOX formulations (6 mg/kg equivalent DOX dose administered on days 1, 8, and 15). All evaluations were performed in a blinded manner by an experienced pathologist, and the corresponding semi-quantitative scoring is summarized in Table 4.

**Table 4:** Semi-quantitative histopathological scoring of tissue damage across organs. Data are presented as Mean ± SD (*n* = 3). Lesion severity was evaluated using a 4-point scoring rubric: Grade 0 = normal, Grade 1 = mild, Grade 2 = moderate, Grade 3 = severe. (Statistical significance was determined using two-way ANOVA followed by Dunnett's post-hoc test. Asterisks indicate statistically significant differences compared to the Control group: *p < 0.05, **p < 0.01$, ***p < 0.0001).

Organ	Control	Free DOX	PDL	F2	F3	F4
Brain	0.0 ± 0.0	2.3 ± 0.3****	0.7 ± 0.3	1.7 ± 0.3****	1.0 ± 0.0	0.7 ± 0.3*
Heart	0.0 ± 0.0	2.0 ± 0.0****	0.3 ± 0.3	0.7 ± 0.3	0.3 ± 0.3	0.3 ± 0.3
Lung	0.0 ± 0.0	1.7 ± 0.3****	1.0 ± 0.0*	0.7 ± 0.3	2.0 ± 0.0****	2.3 ± 0.3****
Kidney	0.0 ± 0.0	1.0 ± 0.0*	1.0 ± 0.0*	1.7 ± 0.3****	2.7 ± 0.3****	1.3 ± 0.3**
Liver	0.0 ± 0.0	0.7 ± 0.3	1.0 ± 0.0*	1.0 ± 0.0*	2.0 ± 0.0****	0.3 ± 0.3
Spleen	0.0 ± 0.0	1.3 ± 0.3**	1.0 ± 0.0*	1.0 ± 0.0*	1.7 ± 0.3****	1.3 ± 0.3**

Histopathological analysis revealed normal brain architecture in the control group (score 0.00 ± 0.00). In contrast, free DOX administration caused marked interstitial edema and diffuse structural disruption (*p* < 0.0001). The PDL group showed only mild focal perivascular inflammation without necrosis. In the F2 group, damage intensified to mild-to-moderate focal-to-multifocal perivascular inflammation and interstitial edema. However, groups F3 and F4 exhibited a more attenuated pattern; while focal inflammation and edema persisted, the lesions were more limited than in F2 (*p* < 0.001), and notably, no necrosis or severe structural damage was observed, indicating reduced neurotoxicity compared to DOX.

The control group exhibited normal myocardial architecture with regularly arranged muscle fibers. Conversely, DOX administration induced mild-to-moderate fiber disorganization and interstitial edema (*p* < 0.01), though without overt necrosis. The PDL group maintained a largely preserved structure, showing only slight interstitial widening. In the F2 group, myocardial stability was mostly retained, with minimal fiber disarray and subtle interstitial separation. Notably, the F3 and F4 groups demonstrated an even more organized pattern; despite minimal interstitial widening, the fibers remained well-aligned with no evidence of inflammatory infiltration or necrosis (p < 0.01), signifying a clear reduction in DOX-induced cardiotoxicity.

Lung sections from the control group showed normal alveolar architecture. DOX administration led to moderate vascular congestion and mild inter-alveolar septal thickening (p < 0.01). In the PDL group, mild septal thickening was accompanied by focal inflammatory infiltration. While the F2 group maintained mild septal thickening without significant inflammation, a progression in pathological changes was noted in the F3 and F4 groups. These groups exhibited moderate-to-severe septal thickening and a distinct reduction in alveolar lumen size; however, despite these structural alterations, no evidence of overt necrosis was observed across all treated groups.

Histological evaluation of renal tissue in the control group showed normal glomerular and tubular architecture. Both DOX and PDL groups exhibited similar mild tubular vacuolization while maintaining overall structure (*p* < 0.05). Injury became more pronounced in the F2 group, characterized by moderate tubular degeneration and focal intratubular casts. The most severe renal alterations occurred in the F3 group, featuring marked tubular degeneration, focal necrosis, and mild interstitial inflammation (*p* < 0.0001). In contrast, the F4 group demonstrated a significant attenuation of these injuries (*p* < 0.001 *vs.* F3); tubular degeneration was limited to a mild-to-moderate range, and notably, no evidence of extensive necrosis was observed, indicating a relative recovery compared to F3.

The control group maintained normal hepatic architecture without inflammatory or degenerative changes. While the DOX group showed only mild periportal degeneration, the PDL and F2 groups exhibited slight inflammatory infiltration and subtle hepatocellular changes. Liver injury peaked in the F3 group, characterized by moderate periportal inflammation and pronounced hepatocellular degeneration (p < 0.0001). Conversely, the F4 formulation effectively preserved the lobular architecture, showing only minimal focal inflammatory cell infiltration and no evidence of extensive tissue damage (p < 0.0001 *vs.* F3), suggesting a superior safety profile compared to F3.

The control group exhibited intact splenic architecture with distinct red and white pulp. In the free DOX group, while the overall structure was preserved, red pulp congestion was accompanied by lymphoid depletion (*p* < 0.01). Conversely, treatments shifted the response toward lymphoid activation; the PDL and F2 groups maintained structural integrity with mild congestion and emerging lymphoid hyperplasia. This response intensified in the F3 and F4 groups, which displayed prominent lymphoid follicles indicative of moderate hyperplasia alongside mild-to-moderate red pulp congestion. Notably, no evidence of necrosis or structural damage was observed in the F4 group, confirming the preservation of splenic integrity under this formulation.

## 4 Discussion

In the current work various formulations were developed using two strategies: negative charge induction (F1–F6) and PEGylation (F7). Post-dialysis DLS analysis confirmed particle sizes within the 100–200 nm range. Danaei et al. categorized this range as ideal for injectable systems (Danaei et al., 2018), while Kaddah et al. noted that formulations outside these limits are generally not classified as nanoliposomes (Kaddah et al., 2018). Particle size increased following doxorubicin DOX loading; however, Ibrahim et al. reported that gradient-based systems typically maintain sizes near 120 nm with low PDI (Ibrahim et al., 2022). Size consistency in F1–F3 and F7 suggests successful loading, whereas substantial increases in F4–F6 likely reflect membrane instability or gradient disruption (Bnyan et al., 2018).

Most formulations maintained desirable PDI values, though F6 exhibited higher heterogeneity. Danaei et al. defined a PDI ≤0.2 as generally acceptable (Danaei et al., 2018), while Fox et al. noted that values exceeding 0.3 may still represent relatively homogeneous populations (Fox et al., 2021). The heterogeneity in F6 coincided with peak Tween 80 concentration. In this regard, Bnyan et al. established that surfactants significantly could modulate vesicle structural properties (Bnyan et al., 2018). Although Witayaudom and Klinkesorn reported improved PDI stability with increasing Tween 80 (Witayaudom and Klinkesorn, 2017), Tao et al. observed increased heterogeneity or reduced zeta potential (Tao et al., 2018). Post-loading PDI increases in F5 and F6 likely reflect broader size distributions or transient aggregates (Ijäs et al., 2021). This aligns with the finding by Bnyan et al. that polysorbates can disrupt bilayer organization through detergent-like effects (Bnyan et al., 2018). Given these membrane-dependent influences the shifts in F5 and F6 are likely attributable to larger subpopulations or aggregation (Roces et al., 2020).

Furthermore, the long-term storage stability evaluation of the lead formulations (F1 and F2) confirmed their robustness, as evidenced by the negligible changes in particle size and PDI over a 3-month period. This stability is crucial for the shelf-life and clinical applicability of these liposomal systems, ensuring that the structural integrity is preserved under storage conditions.

All formulations exhibited negative zeta potentials. Previously it was noted that while negative potentials drive electrostatic repulsion, near-zero values in PEGylated systems like F7 still provide colloidal stability *via* steric stabilization (Liu et al., 2014; Németh et al., 2022). Further, the reduced zeta potential in DSPE-PEG formulations could be attributed to the charge shielding by the hydrated PEG layer (Nakamura et al., 2012; Zhang et al., 2016). Post-loading, the decrease in negative zeta potential likely stemmed from DOX amino group protonation and interactions with anionic lipids (de Wolf, 1991; Peetla et al., 2010). Conversely, Lee et al. found that PEG coating mitigates non-specific interactions by sterically masking the surface charge (Lee et al., 2022). F7's dimensional stability confirms steric effectiveness, consistent with Ibrahim et al. regarding size retention near 120 nm following surface modification (Ibrahim et al., 2022).

TEM confirmed bilayer vesicles consistent with HSPC/cholesterol liposomes. The alignment between TEM and DLS dimensions indicates a stable nanoscale population without significant aggregation, corroborating the low PDI values as observed by Danaei et al. (Danaei et al., 2018).

Phospholipid content was quantified using the Bartlett assay to normalize drug-to-lipid ratios and loading efficiency. This quantification provided a reliable basis for inter-formulation comparisons, preventing errors due to varying lipid concentrations (Bartlett, 1959). DOX was quantified using spectrofluorimetry at excitation/emission wavelengths of 450/585 nm. EE exhibited two distinct trends. Previous reports noted that ammonium sulfate gradient systems typically achieve EEs of 90–99%, where DOX-sulfate nanocrystals stabilize drug retention (Alyane et al., 2016; Haran et al., 1993; Wei et al., 2018). In this study, F2 and F7 achieved EEs within this range, as indicative of successful loading. Calculated values slightly exceeding theoretical boundaries (∼100%) in certain formulations represent minor analytical fluctuations inherent to spectrofluorimetric calibration curves at near-quantitative entrapment levels (Barenholz, 2012). Given the increased size and PDI in F5 and F6, it is suggested that factors driving heterogeneity and enlargement simultaneously compromise loading efficiency (Zhang et al., 2018).

At physiological pH in PBS, most formulations showed gradual release, while F5 exhibited a pronounced initial burst. Gómez-Lázaro et al. associated such elevated release with surface-bound drug or membrane defects (Gómez-Lázaro et al., 2024), consistent with the high heterogeneity observed in F5. Jayaraj et al. further noted that dialysis can accentuate formulation sensitivity, increasing observed release (Jayaraj et al., 2024). In succinate buffer (pH 5.5), release was elevated across all formulations. This pH-dependent enhancement aligns with DOX behavior, where protonation increases free drug solubility and membrane efflux. Wei et al. suggested that acidic conditions also alter membrane permeability, synergistically enhancing diffusion (Wei et al., 2018). Profiles in dextrose-histidine buffer (pH 6.5) indicate that buffer chemistry and ionic species modulate release beyond pH (Gómez-Lázaro et al., 2024). Histidine and sucrose are standard industrial stabilizers for DOX liposomes (Eneli et al., 2025; Niyonshuti et al., 2024; Roces et al., 2020). At pH 4.7, F7 and PDL showed minimal release. Bhadran et al. attributed the stability of PDL to active loading and low-permeability membranes, which improve tumor accumulation (Bhadran et al., 2025). Release variations are often ascribed to membrane microarchitecture and internal drug phase quality (Li et al., 2020). Wei et al. observed that even with similar PEGylation, internal phase variations can alter free drug proportions and release rates (Wei et al., 2018).

Nanoliposomal formulations yielded IC_50_ values between 0.308 and 1.360 μM, with several outperforming PDL (1.965 μg/mL) and rivaling free DOX (0.383 μg/mL). Since prolonged circulation and the EPR effect are irrelevant in cell culture, cytotoxic responses depend primarily on rapid intracellular drug availability (Gabizon et al., 2025). The lower IC_50_ of free DOX compared to PDL is consistent with results from Zaleskis et al. who observed faster cellular and nuclear uptake for free drug *versus* slow-release PEGylated systems (Zaleskis et al., 2021). While PEGylation improves colloidal stability, it can reduce *in vitro* cell adhesion and uptake (Nosova et al., 2019), explaining the higher IC_50_ of PDL. Conversely, the high cytotoxicity of certain nanoliposomes suggests that optimized release and uptake can enhance delivery; Sun et al. similarly reported that nanocarriers can improve intracellular DOX delivery in tumor models (Sun et al., 2017). Furthermore, Norouzi et al. demonstrated that carrier modifications in glioblastoma cell lines can either enhance or attenuate DOX efficacy (Norouzi et al., 2020). Moreover, cytotoxicity evaluation on healthy brain cells demonstrated that while DOX exhibits high non-selective toxicity (IC_50_ = 1.29 μg/mL), the lead formulation F2 significantly spares healthy cells, displaying a markedly higher IC_50_ of 20.67 μg/mL**,** a high biosafety profile further supported by the excellent biocompatibility and lack of intrinsic cytotoxicity (>90% cell viability) observed with empty blank nanoliposomes. Collectively, integrating these *in vitro* and physicochemical findings, specifically particle size stability (142.4 nm, PDI 0.139), high entrapment (>82%), pH-sensitive drug release, favorable biosafety on healthy brain cells, and preserved cytotoxicity (IC_50_ = 0.44 μg/mL), prioritized F2 over unstable (F4–F6) or slow-releasing formulations as the lead candidate for subsequent *in vivo* evaluation.

The biodistribution of all formulations followed a distinct temporal shift from the systemic circulation to specific organ compartments, driven by formulation-dependent pharmacokinetic profiles evaluated in male BALB/c mice (*n* = 3 per group per time point). At 24 h, blood levels of PDL showed a trend toward higher retention compared to free DOX, as PEG coating reduces opsonization and macrophage recognition to prolong circulation (Fu et al., 2025). Conversely, free drug levels were low due to rapid tissue distribution and systemic toxicity, while F4 exhibited a statistically significant reduction in blood concentration compared to DOX, and the intermediate blood concentrations of F2 and F3 could also suggest tissue distribution or macrophage uptake or early drug leakage (Jiang et al., 2024). In highly perfused organs, free DOX and PDL exhibited the highest initial pulmonary concentrations, likely reflecting intravascular content and initial perfusion (Imlimthan et al., 2025). Cardiac exposure at this stage was significantly higher for free DOX, whereas liposomal encapsulation including F2–F4 markedly reduced early cardiac levels (with F2 reaching statistical significance compared to DOX), potentially diminishing ROS-mediated damage and mitochondrial dysfunction (Li et al., 2024; Tang et al., 2025).

Initial accumulation in the reticuloendothelial system and other tissues was largely mediated by surface interactions and innate immunity. At 24 h, hepatic levels were similar across groups, reflecting initial exposure where free drug largely crosses the sinusoidal endothelium for rapid metabolism while Kupffer cells mediate liposomal uptake (Jiang et al., 2024). At 48 h, however, hepatic accumulation diverged; F2 demonstrated elevated hepatic accumulation, showing a statistically significant increase compared to F4, suggesting the interplay between formulation-specific membrane stability and surface modifications. In the spleen, PDL exhibited the highest accumulation at 24 h, significantly exceeding F2 (Dadpour et al., 2022), while at 48 h, PDL maintained significantly higher retention compared to DOX (Mirhadi et al., 2022). Despite PEGylation, opsonins and protein corona formation facilitate recognition by phagocytic CD169 macrophages (Grabowska et al., 2023; Moghimi et al., 2023; Wang et al., 2023a; Wang et al., 2023b). Conversely, initial renal concentrations were comparable between DOX, PDL, and F2, whereas F3 exhibited a significant reduction compared to DOX (Yazdian-Robati et al., 2023), while low brain concentrations confirmed intact BBB integrity under baseline conditions (Bickel, 2005). To ensure accuracy and eliminate intravascular blood artifacts in these low-penetration tissues, systemic transcardial perfusion was thoroughly performed prior to organ harvesting (Betterton et al., 2023).

By 48 h, the formulations transitioned into a redistribution phase characterized by prolonged circulation and cumulative organ exposure. While blood levels of PDL remained the most persistent (Fu et al., 2025; Jiang et al., 2024), brain concentrations revealed notable trends, with F2 demonstrating the highest accumulation and maintaining a statistically significant increase compared to DOX. This suggests that F2 possesses superior potential for enhanced brain penetration in non-tumor models compared to free drug. In the kidneys, F4 demonstrated a statistically significant accumulation compared to DOX at 48 h, whereas F2 and F3 maintained retention levels comparable to PDL (Yazdian-Robati et al., 2023). Surface modifications such as PS80 can further redistribute organ uptake (Huang et al., 2021a; Huang et al., 2021b; Roointan et al., 2024), and in this case, likely facilitated the enhanced brain accumulation observed for F2 across the intact BBB (Wang et al., 2024a; Wang et al., 2024b).

In the late phase, concentrations in highly perfused organs converged or diverged based on membrane stability. Pulmonary concentrations diverged, with F4 showing a statistically significant reduction compared to DOX at 48 h due to drug release and redistribution (Wang et al., 2024a; Wang et al., 2024b). Spleen localization in red pulp macrophages and CD169 subsets likely continues to influence immune responses (Brodin et al., 2025). Cardiac levels remained lower in liposomal formulations (with F2 showing a statistically significant reduction compared to free DOX at 48 h), explaining the reduced toxicity. Finally, hepatic levels diverged by 48 h; F2 levels were highest among liposomal formulations (significantly exceeding F4), reflecting the interplay between macrophage-mediated clearance, membrane stability (Jiang et al., 2024; Kumar et al., 2023), and the role of Tween 80 in modulating distribution pathways (Chen et al., 2024; Ly et al., 2024; Saraswat et al., 2025). Overall, this multi-parametric integration of high baseline brain accumulation (2.78 μg/g tissue at 48 h) alongside a 6-fold reduction in early cardiac exposure confirms F2 as the optimal formulation balancing efficacy and safety.

Histopathological assessment performed in a blinded manner by an independent pathologist (*n* = 5 per group) revealed a fundamental distinction between the direct tissue damage induced by free DOX and the modulated, often macrophage-mediated, responses of the liposomal formulations. Treatment with free DOX resulted in widespread degenerative changes across all major organs. In the heart, high tissue exposure induced myocardial fiber disarray and interstitial edema (Soudi et al., 2025), while in the liver, direct ROS generation and mitochondrial dysfunction led to significant vacuolization and inflammation (El-Dessouki et al., 2025). This toxic profile extended to the kidneys, where tubular vacuolization aligned with established models of oxidative stress (Abd-Ellatif et al., 2022; Radeva and Yoncheva, 2025), and to the spleen, where immunotoxicity caused a marked reduction in lymphoid cellularity (Saleh et al., 2022). Furthermore, free drug-induced ROS triggered pulmonary alveolar wall changes (Subasic et al., 2021) and diffuse cerebral edema *via* BBB compromise (Bhatt et al., 2025; Patel et al., 2025).

In contrast, nanoliposomal encapsulation (PDL and F2–F4) generally preserved tissue architecture by altering pharmacokinetics and limiting nonspecific distribution (Gabizon et al., 2025; Mojarad-Jabali et al., 2025). In the heart, these formulations prevented necrosis and maintained myocardial alignment, particularly in F3 and F4. In the liver and spleen, the response shifted from cellular destruction to mild inflammatory and immune activation (Gabizon et al., 2025). Wang et al. noted that Kupffer cell uptake of PEGylated liposomes often causes mild portal inflammation without altering lobular architecture (Wang et al., 2022), a profile best maintained by formulation F4. Splenic findings for PDL and F2–F4 showed preserved follicles and progressive lymphoid hyperplasia rather than depletion; Wauters et al. attributed this to red pulp macrophage uptake and follicular responsiveness rather than destructive inflammation (Brodin et al., 2025; Wauters et al., 2024).

The severity of localized responses varied significantly based on release kinetics and surface modifications. In the kidneys, while F4 showed limited tubular injury, F2 exhibited moderate degeneration (Chen and Gu, 2023; Lu et al., 2025). Critically, F3 displayed the most severe renal and hepatic profile, including focal necrosis and pronounced degeneration, respectively. Huang et al. linked such peak toxicity to accelerated lysosomal drug release and the activation of apoptotic pathways (Huang et al., 2024). Interestingly, in the lungs, the moderate-to-severe septal thickening in F3 and F4, despite a lack of necrosis, likely reflected transient microvascular trapping and subsequent immune-mediated thickening rather than direct cytotoxicity (Aloss and Hamar, 2023; Jiang et al., 2024).

Finally, neurological responses in the liposomal groups were predominantly vascular-inflammatory rather than cytotoxic (Dias-Carvalho et al., 2024). Wang et al. and Prasad et al. confirmed that Tween 80 facilitates receptor-mediated BBB transport, enhancing nanoparticle interaction with the cerebral vasculature (Prasad et al., 2024; Wang et al., 2025). Gomes et al. suggested that intensity differences in perivascular immune responses reflect variations in protein corona composition as seen in F2 and F3 (Gomes et al., 2025), while others identified perivascular macrophages as the primary drivers of this neuroinflammatory signaling (Ineichen et al., 2022; Zhan et al., 2025).

## 5 Conclusion

The present study addresses the challenge of limited efficacy of systemic chemotherapy in treating brain tumors, primarily due to the restrictive nature of the BBB. Lipid-based nanocarriers, particularly DOX-loaded nanoliposomes incorporating Tween 80, were investigated as a strategy to enhance drug delivery to the CNS while minimizing systemic toxicity. Physicochemical characterization confirmed that Tween 80 influences particle size, zeta potential, and colloidal stability, and formulation adjustments allowed controlled, pH-responsive drug release suitable for acidic tumor-like microenvironments. *In vitro* assays demonstrated significant cytotoxicity and improved antitumor activity in formulations containing Tween 80, highlighting its role in enhancing cellular uptake. Furthermore, cytotoxicity evaluations on normal NIH 3 T3 cells confirmed an excellent biosafety profile, demonstrating that empty blank formulations lack intrinsic toxicity (>90% cell viability) and that the lead formulation F2 significantly spares healthy cells compared to free DOX. Additionally, the observed long-term physical stability confirms the reliability and shelf-life potential of these systems. *In vivo*, the formulations markedly modulated tissue exposure patterns, particularly in the liver and kidneys at 48 h, and reduced systemic toxicity, notably the cardiotoxicity associated with free DOX. Importantly, although overall brain penetration remained limited, the Tween 80–containing formulation F2 achieved significantly higher brain accumulation compared with free DOX, PDL and the other nanoliposomal formulations, indicating an improved potential for crossing the intact blood–brain barrier in baseline models. These findings suggest that Tween 80-containing nanoliposomes provide adjustable drug release, effective cellular delivery, and improved safety profiles, while offering a promising platform for enhancing drug exposure in the brain. However, as these evaluations were conducted in healthy animal models, future studies in orthotopic brain tumor-bearing models remain essential to confirm targeted intratumoral delivery and therapeutic efficacy.

## CRediT authorship contribution statement

**Masoud Khatibi:** Writing – original draft, Investigation, Formal analysis, Data curation. **Seyedeh Maryam Hosseinikhah:** Writing – review & editing, Methodology, Data curation. **Seyedeh Hoda Alavizdaeh:** Writing – review & editing, Supervision, Project administration, Funding acquisition, Conceptualization. **Fatemeh Gheybi:** Validation, Methodology. **Mahmoud Reza Jaafari:** Supervision, Resources. **Zohreh Efati:** Writing – review & editing, Visualization, Formal analysis. **Farzaneh Afshari:** Validation, Investigation, Formal analysis. **Samaneh Yaghoubi:** Methodology, Investigation. **Parviz Norouzi:** Writing – review & editing, Supervision.

## Declaration of competing interest

The authors declare that there is no conflict of interest regarding the publication of this paper.

## Data Availability

Data will be made available on request.
