# Supplementary material for: Unlocking the brain: Biodistribution insights into tween 80 nanoliposomes in healthy brains
Source: Int J Pharm X. 2026 Aug 13;12:100636. doi: 10.1016/j.ijpx.2026.100636 (PMC13524492; doi:10.1016/j.ijpx.2026.100636)
Supplement: Supplementary file 1 [file mmc1.docx]

**Figure S1.** Representative DLS intensity size distribution profiles and corresponding Z-average, polydispersity index (PDI), and peak parameters of dialyzed free nanoliposomal formulations (F1, F3, F4, F5, and F6) prior to active drug loading.

**Figure S2.** Representative DLS intensity size distribution profiles and corresponding Z-average, polydispersity index (PDI), and peak parameters of nanoliposomal formulations (F1, F3, F4, F5, and F6) following active doxorubicin (DOX) loading.

**Figure S3.** *In vitro* cell viability of normal murine fibroblast NIH 3T3 cells treated with empty nanoliposomal formulations (blank F2, F3, and F4) at various equivalent lipid concentrations ranging from 0.31 to 10 µg/mL, assessed by the MTT assay after 48 h. Data are presented as mean ±SD (n=3).
